# Chitosan Composites Synthesized Using Acetic Acid and Tetraethylorthosilicate Respond Differently to Methylene Blue Adsorption

**DOI:** 10.3390/polym10050466

**Published:** 2018-04-24

**Authors:** Thomas Y. A. Essel, Albert Koomson, Marie-Pearl O. Seniagya, Grace P. Cobbold, Samuel K. Kwofie, Bernard O. Asimeng, Patrick K. Arthur, Gordon Awandare, Elvis K. Tiburu

**Affiliations:** 1 Department of Biomedical Engineering, University of Ghana, P.O. Box LG 25, Legon, Ghana; tyaessel@st.ug.edu.gh (T.Y.A.E.); albert.koomson@stu.ucc.edu.gh (A.K.); moseniagya@st.ug.edu.gh (M.-P.O.S.); gracecobbold2016@gmail.com (G.P.C.); skwofie2000@gmail.com (S.K.K.); boasimeng@ug.edu.gh (B.O.A.); 2 Department of Biochemistry, Cell and Molecular Biology, University of Ghana, P.O. Box LG 25, Legon, Ghana; parthur14@gmail.com (P.K.A.); gawandare@ug.edu.gh (G.A.); 3 West Africa Center for Cell Biology of Infectious Pathogens (WACCBIP), University of Ghana, P.O. Box LG 25, Legon, Ghana

**Keywords:** TEOS, methylene blue, chitosan, modelling, cross-linking, interpenetrating, XRD, FTIR

## Abstract

The sol-gel and cross-linking processes have been used by researchers to synthesize silica-based nanostructures and optimize their size and morphology by changing either the material or the synthesis conditions. However, the influence of the silica nanostructures on the overall physicochemical and mechanistic properties of organic biopolymers such as chitosan has received limited attention. The present study used a one-step synthetic method to obtain chitosan composites to monitor the uptake and release of a basic cationic dye (methylene blue) at two different pH values. Firstly, the composites were synthesized and characterized by Fourier Transform Infrared Spectroscopy (FTIR) and X-ray Diffraction (XRD) to ascertain their chemical identity. Adsorption studies were conducted using methylene blue and these studies revealed that Acetic Acid-Chitosan (AA-CHI), Tetraethylorthosilicate-Chitosan (TEOS-CHI), Acetic Acid-Tetraethylorthosilicate-Chitosan (AA-TEOS-CHI), and Acetic Acid-Chitosan-Tetraethylorthosilicate (AA-CHI-TEOS) had comparatively lower percentage adsorbances in acidic media after 40 h, with AA-CHI adsorbing most of the methylene blue dye. In contrast, these materials recorded higher percentage adsorbances of methylene blue in the basic media. The release profiles of these composites were fitted with an exponential model. The R-squared values obtained indicated that the AA-CHI at pH ~ 2.6 and AA-TEOS-CHI at pH ~ 7.2 of methylene blue had steady and consistent release profiles. The release mechanisms were analyzed using Korsmeyer-Peppas and Hixson-Crowell models. It was deduced that the release profiles of the majority of the synthesized chitosan beads were influenced by the conformational or surface area changes of the methylene blue. This was justified by the higher correlation coefficient or Pearson’s *R* values (*R* ≥ 0.5) computed from the Hixson-Crowell model. The results from this study showed that two of the novel materials comprising acetic acid-chitosan and a combination of equimolar ratios of acetic acid-TEOS-chitosan could be useful pH-sensitive probes for various biomedical applications, whereas the other materials involving the two-step synthesis could be found useful in environmental remediation of toxic materials.

## 1. Introduction

Biopolymers are macromolecules produced by living organisms; these include chitin, gelatin, cellulose, and a plethora of other diverse chemical entities. The properties of macromolecules are usually modified to make them suitable for most in vivo and in vitro studies [1,2]. Chitosan, the deacetylated derivative of chitin, is a linear cationic biopolymer of glucosamine residues, specifically *N*-acetyl-d-glucosamine and *N*-d-glucosamine, linked through β-(1–4)-glyosidic bonds. Due to its abundance, biocompatibility, and low cost, chitosan has been extensively used either in its natural form or as a derivative with other organic or inorganic molecules in various applications including bioseparation, drug delivery systems, environmental remediation, and imaging [3,4].

However, the high crystallinity, minimum surface area, high molecular weight, excessive swelling, and the degree of acetylation of chitosan impact negatively on its solubility, material forming capacity, biodegradability, and diverse bioactive attributes [5]. In addition, organic-based materials such as chitosan possess reactive functional groups including amines and hydroxyls, which are sites for attachment of other macromolecules and metal ions. For decades, efforts have been targeted at the modification of these biopolymers with suitable molecules for a wide range of applications [6]. For example, chemical cross-linkers such as acetic acid, glutaraldehyde, epichlorohydrin, and tetraethylorthosilicate (TEOS) have been used as alternatives to modify the structures of certain biopolymers including chitosan and, as a result, new functional materials with improved physicochemical properties have been produced [7]. Although these cross-linkers improve the mechanical strength, thermal stability, swelling ability, and pH sensitivity of the chitosan nanocomposite materials, glutaraldehyde, for example, tends to polymerize upon addition to the reaction mixture leading to loss of adsorption sites, thus diminishing the capacity of the resulting end product to be used in drug uptake and delivery [8]. Besides this, most cross-linkers are toxic and their usage in most biomedical applications is limited [9].

The sol-gel process has been used by researchers to synthesize silica nanostructures and optimize their size and morphology by changing either the materials or synthesis conditions [10,11,12]. It has been established that a mixture of an inorganic strong acid such as TEOS with a weak organic acid (acetic acid) can produce nanostructures with unique properties [13]. For example, chitosan can be cross-linked with TEOS to form an interpenetrative network (IPN), thus enhancing drug permeation as well as controlled release of food nutrients. Similarly, chitosan, when mixed with acetic acid, enhances gelation as well as drug entrapment. A hybrid polymer derived from siloxane and chitosan has also been obtained by the sol-gel technique using tetraethylorthosilicate (TEOS) as a precursor to immobilize enzymes for studying a wide range of applications including bio-sensing and bio-catalysis [14,15,16].

However, the influence of the silica nanostructures on the overall physicochemical properties of biopolymers such as chitosan has received limited attention. In most reaction conditions, a two-step reaction scheme is adopted: chitosan is first activated by acetic acid, followed by cross-linking with a suitable molecule [6,7,10,17]. The underlying question that the current work seeks to investigate is whether a one-step reaction pathway, using a mixture of equimolar concentrations of precursor molecules such as TEOS and acetic acid, can influence the overall structure and properties of the parent chitosan molecule. The present study therefore reports a one-step synthetic method of using chitosan composites to monitor the uptake and release of methylene blue at two different pH values, with a combination of equimolar mixture of acetic acid and TEOS. The materials’ adsorption and release capacities of methylene blue is compared to the results of similarly synthesized chitosan composites using a two-step method at the acidic and basic conditions. Methylene blue was used as a model cationic dye to monitor the physicochemical properties of the materials because it has been established that anionic and cationic dyes, have differential properties depending on the pH conditions of the media. This work seeks to propose that the dye adsorptive properties towards the chitosan composites are dependent on pH conditions [18,19,20].

## 2. Materials and Methods

### 2.1. Materials

All analytical-grade chemicals were purchased from Sigma-Aldrich, St. Louis, MO, USA with the exception of TEOS, which was obtained from Philip Harris Ltd., Birmingham, UK. Chitosan (medium molecular weight *M*_w_ = 141 kDa, and degree of acetylation DA = 15–25%) was used as the parent biomolecule for the formation of the composites. Tetraethylorthosilicate (TEOS, 98% grade), a strong inorganic acid, and glacial acetic acid, a weak organic acid, were used as a cross-linker and an activator, respectively, to modify the chitosan.

### 2.2. Methods

#### 2.2.1. Synthesis of Chitosan Beads Using Acetic Acid

Chitosan beads were synthesized with acetic acid according to methods described in previous publications [21,22] with slight modifications.

#### 2.2.2. Synthesis of Chitosan Beads Using TEOS

Chitosan beads were synthesized with TEOS according to the above modified method [6]. Briefly, 2 g of chitosan was added to 2% *v/v* TEOS (2 mL TEOS, 6 mL 0.5 M HCl, and 92 mL deionized water) and the resulting mixture stirred until a uniform solution was obtained. The resulting gel was aspirated with a syringe and added in drops to 250 mL of 2 M NaOH solution. The beads formed in the NaOH solution were stirred at room temperature for 24 h using a magnetic stirrer. The beads were then filtered with deionized water until a neutral pH was achieved and then air dried.

#### 2.2.3. Synthesis of Chitosan Beads Using Double Cross-Linking (Acetic Acid Followed by TEOS)

Chitosan beads were synthesized with acetic acid followed by TEOS according to the established method [23].

#### 2.2.4. Synthesis of Chitosan Beads Using One-Step Method (Equimolar Concentrations of Acetic Acid and TEOS)

The chitosan beads were produced using the sol-gel method with slight modifications [17]. Briefly, 50 mL of 2% *v/v* acetic acid was added to 50 mL of 2% *v/v* TEOS (1 mL TEOS, 3 mL 0.5 M HCl, and 46 mL deionized water). A quantity of 2 g of chitosan was then added and the mixture was stirred until a uniform solution was obtained. The resulting gel was aspirated and added in drops to 250 mL of 2 M NaOH solution. The beads formed in the NaOH solution were stirred at room temperature for 24 h using a magnetic stirrer. The beads were then filtered with deionized water until a neutral pH was reached, and then air dried.

#### 2.2.5. Sample Characterization

A PANanalytical Empyrean X-ray Diffractometer, Almelo, The Netherlands with Cu Kα (λ = 1.5406 Å) was used to acquire the XRD patterns for chitosan and its derivatives. The scan rate was 2° per minute in a range of 5–90°. The FTIR spectra were acquired over the region of 500–4000 cm^−1^ using a PerkinElmer spectrometer, Waltham, Massachusetts, USA. The FTIR scans were processed using the PerkinElmer Spectrum Version 10.03.09.I.

### 2.3. Analysis

#### 2.3.1. Point of Zero Charge

The point of zero charge of each composite was estimated by using an established drift method [24]. Briefly, a fixed amount of each of the synthesized beads was added to 10 mL of deionized water with varying pH values of 2, 4, 6, 8, 10, and 12. The samples were left to sit for 48 h after which pH readings were taken and a graph of the two pH values was recorded. The points of intersection of the two pH readings for each sample were recorded.

#### 2.3.2. Swelling Ratio

Fixed amounts of the various synthesized beads were immersed in separate 10 mL buffers (pH ~ 2.6 and 7.2) and left overnight. The swelling ratio was calculated using the following equation by Park et al. [6].

(1) $$\mathrm{Swelling}\;\mathrm{ratio}=\frac{\mathrm{Weight}\;\mathrm{of}\;\mathrm{wet}\;\mathrm{or}\;\mathrm{swelled}\;\mathrm{beads}-\mathrm{Weight}\;\mathrm{of}\;\mathrm{dried}\;\mathrm{beads}}{\mathrm{Weight}\;\mathrm{of}\;\mathrm{dried}\;\mathrm{beads}}$$

The following equation was used to estimate the %ε (percentage porosity) as described by Chatterjee et al. [5]:

(2) $$\%\varepsilon \;=\;\frac{\left(W_{W}-W_{D}\right)/\rho_{W}}{\frac{W_{D}}{\rho_{\mathrm{Mat}}}\;+\;\left(W_{W}-W_{D}\right)/\rho_{W}}\times 100\%$$

where $W_{W}$ is the weight of the wet beads in grams before drying; $W_{D}$ is the weight of the dry beads in grams; $\rho_{W}$ is the density of water, 1.0 g/mL; and $\rho_{\mathrm{Mat}}$ (g/mL) is the material density of the dry bead.

#### 2.3.3. Methylene Blue Adsorption and Release Properties of the Composites

Diluted methylene blue solutions adjusted to pH ~ 2.6 and 7.2 were prepared from 1 g/L stock methylene blue solution. Adsorbance values were recorded using a JENWAY, 6705 UV-Vis Spectrophotometer, Stone, Staffordshire, UK, at a wavelength of 661 nm. A quantity of 1.5 mL of the diluted dye was added separately to 50 mg of chitosan beads. The resulting solution was gently agitated for even dispersion of beads in the dye. The solution was left to stand for 16, 20, 24, or 40 h, after which 1 mL of the supernatant was used for UV-Vis adsorbance readings. Percentage changes in adsorbances at each time interval were calculated using the formula

(3) $$\%\;\mathrm{change}\;\mathrm{in}\;\mathrm{adsorbance}\;\left(\mathrm{methylene}\;\mathrm{blue}\right)=\;\frac{A_{t}-A_{o}}{A_{o}}\times 100$$

where *A*_o_ and *A*_t_ are adsorbances at times 0 and *t*, respectively.

The release studies were estimated over 240 min using the adsorbent capacity formula as used by Chatterjee et al. [5]:

(4) $$q\;=\;\frac{\left(C_{o}-C_{\mathrm{eq}}\right)}{W}\times V$$

where *q* is the adsorbent capacity, mg/g; *C*_o_ is the initial concentration of methylene blue, g/L; *C*_eq_ is the final or equilibrium concentration of methylene blue, mg/L; *V* is the volume of the experimental solution, *L*; and *W* is the dry weight of the hydrogel beads, g. An exponential fit was used to analyze the best release profile. Models used to describe the release mechanism were Korsmeyer-Peppas and Hixson-Crowell models [25].

## 3. Results

The functionalization of chitosan was undertaken using two cross-linkers at room temperature, and the reaction scheme is outlined in Table 1. The key difference between AA-CHI-TEOS and AA-TEOS-CHI is that the later was generated using a one-step scheme.

**Figure 1:**
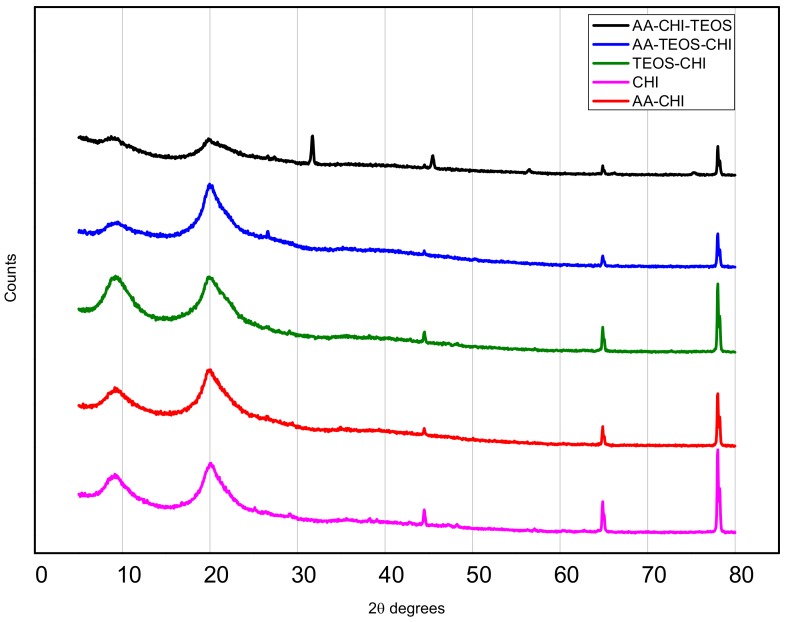
XRD patterns of chitosan and its derivatized composites obtained from acetic acid and TEOS.

In Figure 1, the X-ray diffraction patterns of the chitosan and its synthesized composites from acetic acid and TEOS using the different synthetic routes are displayed. The signature peaks of chitosan (CHI) revealed two unique broad peaks at 2θ degrees at positions 9.1° and 20.1°, indicating the presence of chitosan [26,27,28]. The additional peaks at 2θ degrees of 44.5, 64.9, and 78.0 are probably residual chitin peaks because of the incomplete deacetylation of the parent chitin. Except for AA-CHI-TEOS, all the other composites have similar XRD patterns. Also, additional peaks appeared at about 2θ degrees of 32, as well as the reduction of the background (broad peaks) in the AA-CHI-TEOS pattern, indicating the introduction of a crystalline phase into the sample.

**Figure 2:**
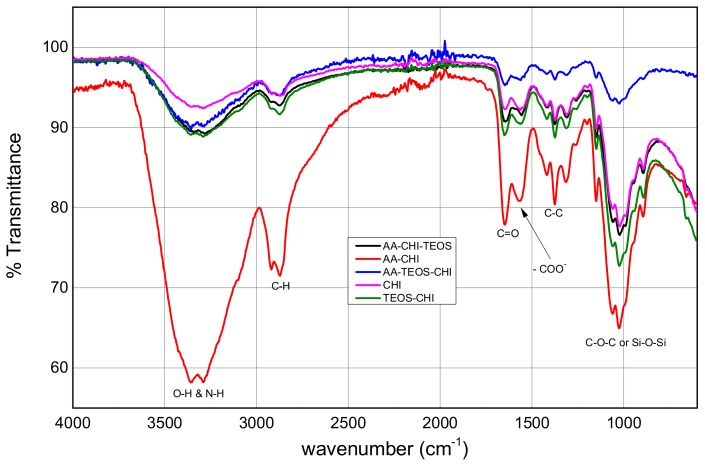
FTIR spectra of pure chitosan and its derivatized composites obtained from acetic acid and TEOS.

The FTIR spectra were obtained within the range of 500–4000 cm^−1^, but the major fingerprints of chitosan as shown in Figure 2 are between 1000 and 3345 cm^−1^. Chitosan and its derivatives all showed broad percentage transmittances greater than 3000 cm^−1^ due to the stretching vibrations of the O–H and N–H bonds. The relative wavenumbers remained unchanged, but the percentage transmittances of the broad peaks differed based on the treatment of the parent chitosan with the various additives. The doublet peaks at 2921/2874 cm^−1^ are characteristic fingerprints of C–H due to symmetric and antisymmetric stretching vibrations. It is also interesting to note that the peaks below 2000 cm^−1^ have different percentage transmittances with AA-CHI having the highest, indicating that acetic acid treatment of chitosan was very efficient in cross-linking the organic additives with chitosan. Except for TEOS-CHI, all the composites showed higher percentage transmittances at wavenumbers below 2000 cm^−1^ compared with the pure chitosan. When chitosan was treated with only TEOS as in the TEOS-CHI sample, the percentage transmittances of the peaks seemed to diminish. The wavenumbers in the IR spectra of pure chitosan and the derivatized product clearly indicated that the parent chitosan was not chemically different from the product but rather that its percent transmittance was influenced by the organic acids.

The points of zero charge (pH_pzc_) of each of the materials as well as their water swelling capacities were estimated at different pH values; the results are displayed in Table 2. The percentage porosities (%ε) of all the synthesized materials are recorded in Table 2. Almost all the samples at pH = 2.6 revealed similar porosities of 86%, 87%, and 88% for AA-CHI, AA-CHI-TEOS, and AA-TEOS-CHI, respectively, except for TEOS-CHI which recorded a %ε of 92. However, different %ε values were obtained at pH = 7.2 for AA-CHI (83%), AA-TEOS-CHI (70%), AA-TEOS-CHI (77%), and TEOS-CHI (89%).

**Figure 3:**
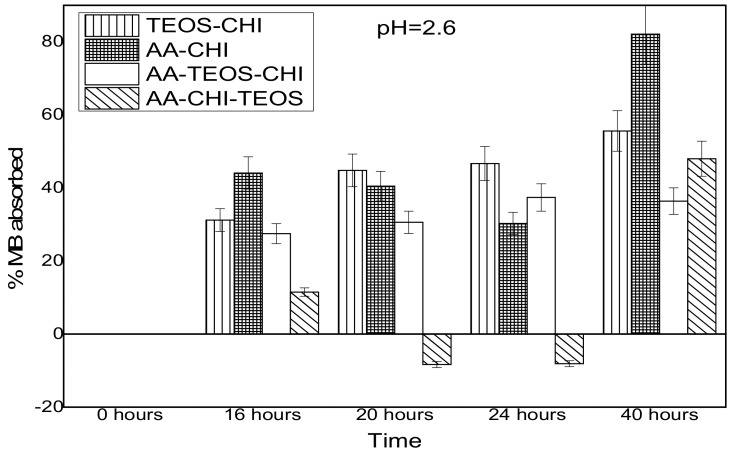
Adsorbance profile of methylene blue at pH = 2.6 as a function of time (hours).

**Figure 4:**
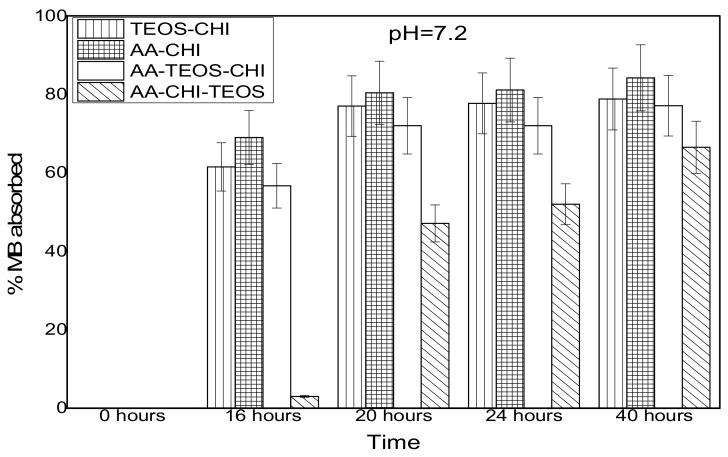
Adsorbance profile of methylene blue at pH = 7.2 as a function of time (hours).

**Figure 5:**
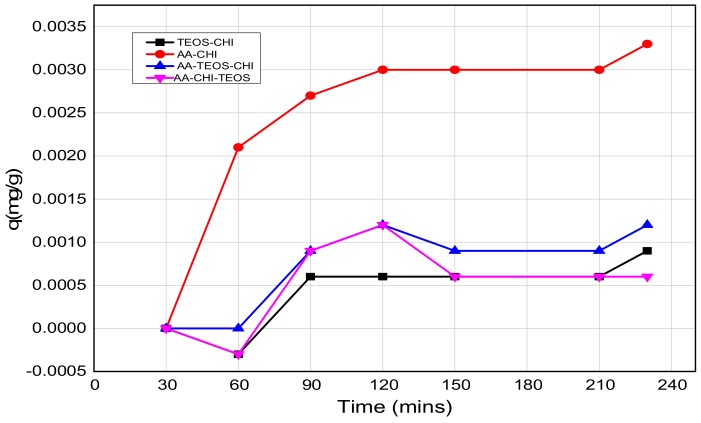
The release profiles of methylene blue at pH = 2.6 as a function of time.

**Figure 6:**
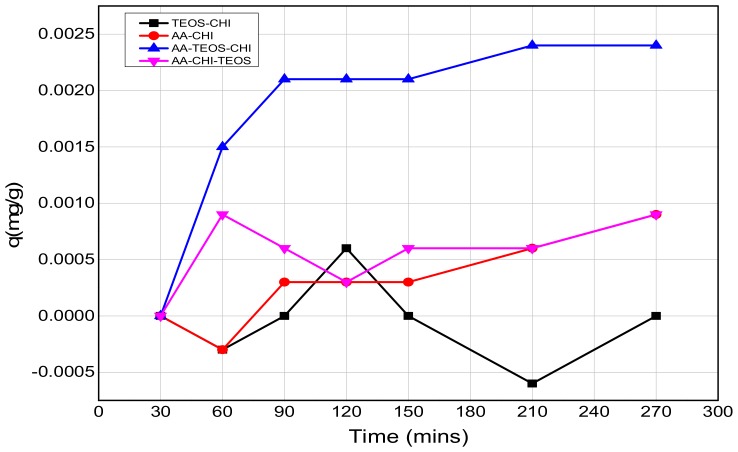
The release profiles of methylene blue at pH = 7.2 as a function of time.

The effect of pH on the adsorption behavior of methylene blue was investigated in both acidic and basic media in different time frames; the results are as shown in Figure 3 and Figure 4. It was observed that after 16 h of adsorption, the trend seemed to favour AA-CHI, with AA-CHI-TEOS exhibiting the least adsorption at pH = 2.6, as shown in Figure 3. After 20 h, TEOS-CHI performed best among all the materials studied. AA-CHI-TEOS recorded a net negative percentage, indicating water uptake from the methylene blue solution. Similar trends were observed after 24 h with slight variation in the adsorption behavior between AA-CHI and AA-TEOS-CHI. The pattern seemed to favour TEOS-CHI, with AA-CHI-TEOS exhibiting the least adsorption behavior at pH = 2.6, as shown in Figure 3. However, after 40 h, all the materials, including AA-CHI-TEOS, showed significant amounts of dye adsorption. It was concluded that after 40 h, AA-CHI had a better adsorption capacity among all materials studied at pH = 2.6. Unlike at the acidic pH conditions, however, there was an overall increase in dye adsorption at pH = 7.2 with abrupt changes to the adsorption behavior of AA-CHI-TEOS after 40 h, as shown in Figure 4. The pH effect on the methylene blue released was also monitored at different time intervals, as shown in Figure 5 and Figure 6.

**Figure 7:**
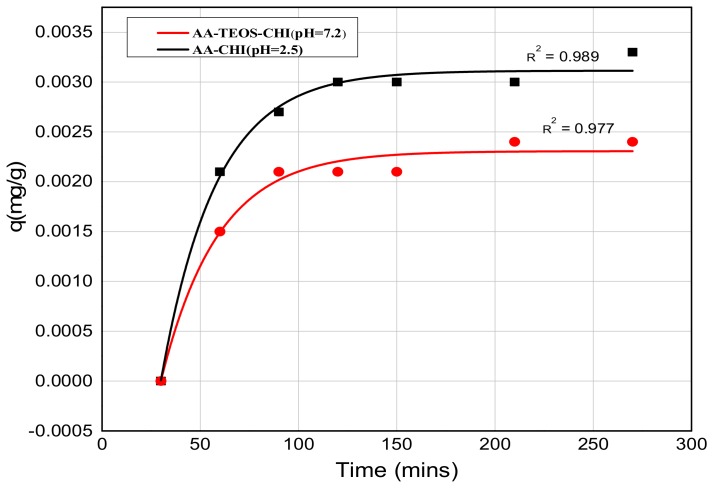
The exponential fit release profile of methylene blue from chitosan composites at two different pH values. The experimental and fitted values are represented by the scatter plots and the solid lines, respectively.

The release profile plots of methylene blue over 240 min are shown in Figure 5 and Figure 6 at pH = 2.6 and 7.2, respectively. At pH = 2.6, the release profile of AA-CHI follows an exponential order whereas the other materials fluctuate over the same time, as shown in Figure 5. However, at pH = 7.2, AA-TEOS-CHI exhibited a unique exponential order as opposed to the other materials as shown in Figure 6. It is interesting to note that AA-CHI, which exhibited progressive exponential order at a lower pH value in Figure 5, now behaves erratically at high pH conditions, and vice versa for AA-TEOS-CHI. This could be due to changes in the pore structures either through shrinkage in the pore size or the modification of certain functional groups within the pore matrix under different pH conditions. An exponential curve fitting model was used to extract *R*-squared values for comparison, as shown in Table 3. As observed, AA-CHI performs best, revealing *R*^2^ values at pH = 2.6 and 7.2 of 0.989 and 0.757, respectively. Similarly, AA-TEOS-CHI also showed reasonable *R*^2^ values of 0.658 and 0.977 at acidic and basic pH, respectively. Thus, the other functionalized materials could not follow the exponential order profile and were subjected to other mathematical models to explain whether the dye conformational properties could influence the materials’ behavior. Nonetheless, as shown in Figure 7, AA-CHI released the dye efficiently at lower pH, whereas AA-TEOS-CHI performed better at higher pH with significant degree of accuracy based on the exponential fit model.

## 4. Discussion

The X-ray diffraction patterns revealed the same positions but different intensities, which probably suggests the introduction of crystalline phase into the chitosan when the biopolymer was treated with the acids. This was especially true for AA-CHI-TEOS, where the background broad peaks had reduced drastically, leading to the appearance of additional peaks. The differences in the FTIR transmittance clearly indicated the modification of the parent chitosan through amide formation and other physicochemical changes caused by the two acids. To facilitate the interpretation of the materials under consideration, parameters such as pH_pzc_ and percentage porosity were carefully determined in order to provide the surface charge and porosity information that are crucial determinants of material behavior. All the materials synthesized except for TEOS-CHI were neutral in acidic media based on the point of zero charge (pH_pzc_) values. In similar acidic conditions, methylene blue also existed as a cationic molecule and, as a result, only simple diffusion of methylene blue would be permissible for the dye uptake into these materials. However, approximately 30–45% of methylene blue had permeated AA-TEOS-CHI and AA-CHI-TEOS after 40 h, whereas within the same time frame, AA-CHI with comparable pH_pzc_ and %ε (% porosity) recorded almost 80% methylene blue adsorption. These observations underscored the importance of surface charge neutralization and porosity, which are the major determinants of dye adsorption into these materials. As a result, we attributed this behavior to the influence of the silica framework in the adsorption behavior of AA-TEOS-CHI and AA-CHI-TEOS. As previously reported, the swollen state of chitosan within the silica nanostructures at high pH, the insolubility of the chitosan composite, and the barrier created by the silica framework could impact the dye’s diffusion into the interpenetration polymer pore network of these two materials, causing a significant reduction in methylene blue’s uptake [6]. Such physicochemical factors were not available in AA-CHI to impede the simple diffusion of the dye into the activated chitosan. Based on the observations, it was speculated that inherent properties requiring energy for active transport of the methylene blue into AA-CHI-TEOS could be the driving force for the low uptake of the dye. This was especially true for AA-CHI-TEOS where water uptake was the preferred choice for the material as indicated in the negative adsorption profile.

At basic pH, there was rapid response (70–80%) in methylene blue uptake by all the materials studied after 40 h. It was observed that the percentage porosity of the chitosan composites drastically reduced in basic pH due to shrinkage of the chitosan within the silica framework, yet dye uptake was significantly higher compared with the values at high pH (acidic medium). This could be due to the biopolymer shrinkage within the silica framework that might be responsible for the elevated amount of dye uptake at low pH (basic medium). Again, AA-CHI seems to have the highest adsorption capacity among all the materials because of increased surface area at low pH.

To probe the release kinetics of entrapped methylene blue at the two pH conditions, the release isotherm model was used to monitor the release of the dye as a function of time in phosphate-buffered saline. The release kinetics of the entrapped methylene blue were evaluated using the Korsmeyer-Peppas ($\frac{M_{t}}{M_{\infty }}=Kt^{n}$) model, where $\frac{M_{t}}{M_{\infty }}$ is the fraction of the dye released at time *t*, *K* is the release rate constant, and *n* is the release exponent. The *n* values corresponding to the released mechanism of the dye from the material at different pH values were determined. The boundary conditions set by the above model to determine the type of release mechanisms of the dye were based on the *n* values. From our determination, the computed *n* values from this work concluded that the transport mechanism did not follow the Fickian diffusion mechanism, or other non-Fickian transport such as the Case II transport or Super Case II transport.

Our attention was then directed towards using the Hixson-Crowell Model to evaluate whether conformation changes of the dye could have an influence on the release process. The formula used to evaluate this assertion is given by ${C_{o}}^{\frac{1}{3}}-{C_{t}}^{\frac{1}{3}}=K_{\mathrm{HC}}t$, where *C_o_* = initial amount of drug adsorbed, *C*_t_ = amount of drug remaining at time *t*, and *K*_HC_ = rate constant. A graph of (${C_{o}}^{\frac{1}{3}}-{C_{t}}^{\frac{1}{3}}$) was plotted against *t* for the release profile of each composite synthesized and Pearson’s *R* was recorded based on the linear fit constructed for each release profile. The boundary conditions for this model were that if the absolute value of the correlation coefficient or Pearson’s *R* of the above equation was higher, then the release mechanism was as a result of a change in surface area/conformation of the dye. The Pearson’s *R* for the release profile of each chitosan bead is reported in Table 4. From the table, it was concluded that change in surface area/conformation of the dye has a significant effect on the respective release profiles of AA-CHI, AA-TEOS-CHI, and AA-CHI-TEOS at pH = 2.6, while in the slightly basic pH, the release of TEOS-CHI, AA-CHI, and AA-TEOS-CHI was also strongly influenced by the change in surface area or conformation of the dye.

However, it is important to note that the steady rise in the release profile just before the plateau region suggests an asymptotic behavior which is due to the exponential release pattern. In the acidic medium, only AA-CHI revealed a release profile that follows an exponential order but none of the materials’ behaviors were able to fit that model. As expected, the adsorption studies in acidic media wherein AA-CHI entrapped most of the dye after 40 h followed an exponential release curve with an *R*^2^ value close to 1. The other composites at the same pH exhibited a release profile deviant from the exponential model and this is evident from the low *R*^2^ values. In terms of the release profile in basic medium, AA-TEOS-CHI followed an exponential profile reminiscent of what is observed at pH = 2.6 for AA-CHI. In terms of the practical applications of the materials synthesized, AA-CHI and AA-TEOS-CHI could be useful for wider biomedical applications including drug entrapment and release due to their steady mechanistic behavior, whereas the other materials could find use in environmental remediation of toxic materials.

## 5. Conclusions

The current study highlights a unique opportunity to utilize a combination of organic and inorganic precursors in either a one-step or two-step reaction mechanism to produce new functional materials that could either be used for targeted drug delivery or for environmental remediation. It has been revealed that two of the materials comprising acetic acid-chitosan and a combination of equimolar ratios of acetic acid-TEOS-chitosan could be useful pH-sensitive probes for various biomedical applications, whereas the other materials from the two-step synthesis could be useful in environmental remediation of toxic materials.

## Figures and Tables

**Table 1:** Different reaction schemes for the synthesis of chitosan composites.

	Chitosan (CHI)	Acetic acid (AA)	TEOS
CHI	√		
AA-CHI	√	√	
TEOS-CHI	√		√
^a^ AA-CHI-TEOS	√	√	√
^b^ AA-TEOS-CHI	√	√	√

**^a^** AA-CHI-TEOS are beads synthesized by first activating chitosan with acetic acid and cross-linked with tetraethylorthosilicate (TEOS). ^b^ AA-TEOS-CHI are beads which were synthesized with a mixture of equimolar concentrations of both acetic acid (weak organic acid) and TEOS (strong inorganic acid).

**Table 2:** Swelling studies conducted at different pH values. The point of zero charge (pH_pzc_) of each sample is also indicated.

Sample	Swelling ratio		% Porosity (%ε)		pH_pzc_
	pH = 2.6	pH = 7.2	pH = 2.6	pH = 7.2	
AA-CHI	3.0	2.4	86	83	7.6
TEOS-CHI	4.0	2.6	92	89	3.5
^a^ AA-CHI-TEOS	4.0	2.0	87	77	7.1
^b^ AA-TEOS-CHI	3.0	1.0	88	70	7.5

**^a^** AA-CHI-TEOS are beads synthesized by first activating chitosan with acetic acid and cross-linked with tetraethylorthosilicate (TEOS). ^b^ AA-TEOS-CHI are beads which were synthesized with a mixture of equimolar concentrations of both acetic acid (weak organic acid) and TEOS (strong inorganic acid).

**Table 3:** A table of R-squared values obtained from an exponential fit of the release profile.

Samples	*R*^2^ Values	
	pH = 2.6	pH = 7.2
AA-CHI	0.989	0.757
TEOS-CHI	0.539	−0.5
^a^ AA-CHI-TEOS	0.156	0.380
^b^ AA-TEOS-CHI	0.658	0.977

**^a^** AA-CHI-TEOS are beads synthesized by first activating chitosan with acetic acid and cross-linking with tetraethylorthosilicate (TEOS). ^b^ AA-TEOS-CHI are beads which were synthesized with a mixture of equimolar concentrations of both acetic acid (weak organic acid) and TEOS (strong inorganic acid).

**Table 4:** Evaluation of the release mechanism of methylene blue using Hixson-Crowell model.

Sample		TEOS-CHI	AA-CHI	AA-TEOS-CHI	AA-CHI-TEOS
Pearson’s *R*	pH = 2.6	−0.18	0.92	0.76	0.52
	pH = 7.2	0.77	0.745	0.748	0.38
